# Assessment of endogenous calcium loss from different sources in 45-week-old Hy-Line Brown laying hens

**DOI:** 10.5713/ab.25.0008

**Published:** 2025-05-12

**Authors:** Nuwan Chamara Chathuranga, Shan Randima Nawarathne, Elijah Ogola Oketch, Venuste Maniraguha, Bernadette Gerpacio Sta. Cruz, Jeseok Lee, Haeeun Park, Hyunji Choi, Myunghwan Yu, Jung Min Heo

**Affiliations:** 1 Department of Animal Science and Biotechnology, Chungnam National University, Daejeon, Korea

**Keywords:** Apparent, Calcium, Endogenous Loss, Excretory, Ileum, Standardized

## Abstract

**Objective:**

The study assessed the calcium (Ca) endogenous losses and digestibility in 45-week-old Hy-Line Brown laying hens fed diets incorporating different Ca-sourcing ingredients.

**Methods:**

A total of 168 hens were randomly assigned to dietary treatments, with six replicates, and four hens were housed per cage. The seven diets included different Ca sources of monocalcium phosphate (MCP), dicalcium phosphate (DCP), limestone, corn, soybean, wheat bran, and a Ca-free diet. All diets included 0.3% Cr_2_O_3_ as a digestible marker as well. Hens were given *ad-libitum* access to feed and water. On day 3, fresh excreta and the ileal digesta were collected to analyze dry matter, Ca, and Cr_2_O_3_ for digestibility analysis.

**Results:**

The Ca-free diets led to lower endogenous Ca losses at the ileum (p<0.05) compared to the Ca-supplemented groups. Among the Ca-sourcing diets, MCP resulted in higher Ca losses, whereas corn-based diets showed relatively lower losses at the ileum. Calcium losses at the excretory site were not significant, although the ileal losses were markedly higher (p<0.001), with diet-by-site interaction (p<0.05). Apparent and standardized ileal Ca digestibility were higher for inorganic sources (MCP, DCP, limestone) than for plant-based sources, with DCP showing greater digestibility (p<0.05).

**Conclusion:**

Inorganic Ca sources resulted in increased endogenous Ca loss and digestibility compared to plant seed-based layer diets, with these losses varying depending on the specific measurement sites.

## INTRODUCTION

Generally, plant-based diets for laying hens are supplemented with inorganic sources, such as limestone, mono, and dicalcium phosphates (DCPs), due to their widespread availability and effectiveness in meeting calcium (Ca) requirements [1]. Besides those dietary Ca sources, several origins contribute to endogenous Ca in the gastrointestinal tract of chickens, including saliva, gastric secretions, biliary and pancreatic secretions, and shed gastrointestinal cells [2]. Calcium loss through internal sources is referred to as “endogenous calcium loss.” This loss is measured at the terminal ileum, assuming that the unabsorbed portion beyond this point is fundamentally lost to the animal. There are two categories of endogenous losses: basal endogenous losses, which occur independently of the diet, and specific endogenous losses, which are influenced by the diet. Consequently, the total endogenous loss of nutrient is the combined sum of both basal and specific endogenous losses [3]. The estimation of this lost amount is an essential part of calculating the standardized ileal or true ileal digestibility of Ca [4].

Calcium is the most prominent mineral in the animal skeletal system [1]. It is involved in blood coagulation, muscle contraction, nerve impulse transmission, hormone secretion, enzyme activation, and cardiac regulation as a physiological function in animals [5,6]. In addition, Ca participates in numerous biological processes including skeletal development, egg formation, and energy metabolism in layers [7]. Furthermore, Ca concentrations have been found to directly influence the digestion and absorption of phosphorus [8], nitrogen [9], and lipids in chickens, although carbohydrates remain unaffected [4,10].

Layers demand much elevated Ca levels than non-layers during the laying periods for production purposes [8]. For instance, when 4 g of calcium is ingested by a hen, approximately 0.5 g is excreted in the feces, around 0.4 grams are eliminated through the urine, and 0.1 grams are incorporated into the bone matrix. Thus, roughly 3 g of Ca is available for egg formation and of this, 2 g is deposited in the eggshell, while the remaining calcium is allocated to the yolk and albumin [11]. Hence, egg formation demands higher plasma Ca levels and, if the dietary Ca is insufficient, hens mobilize calcium from bones [7]. This process can lead to skeletal disorders such as osteoporosis and cage layer fatigue in chickens [12]. Therefore, ensuring a well-optimized Ca provision is essential for the health and productivity of hens in the layer industry. This study investigated endogenous Ca loss in 45-week-old Hy-Line Brown laying hens and evaluated the standardized ileal digestibility (SID) and the apparent ileal digestibility (AID) of Ca across seven different Ca-sourcing diets.

## MATERIALS AND METHODS

### Birds, housing, and management

A total of 168, 45-week-old Hy-line Brown laying hens were used in this study. All hens were fed standard layer diets until the start of the study when they were weighed individually and randomly assigned to experimental diets in a completely randomized design with four birds per cage and six replicates per dietary treatment. All birds had access to feed and water on an *ad libitum* basis. Each cage (60×25×45 cm^3^) was equipped with two nipple drinkers and a metal feeder. The lighting program employed 16 hours of continuous light and 8 hours of darkness in a windowless and temperature-controlled facility (around 20°C to 22°C). The study followed the guidelines of the Hy-Line Brown management guide [13].

### Diets

Table 1 shows the ingredients and chemical compositions of the diets fed for three days. The test ingredients included monocalcium phosphate (MCP), DCP, limestone, corn, soybean meal, and wheat bran as the sole source of calcium. A Ca-free diet was subsequently formulated to estimate the basal endogenous Ca losses. All seven diets contained 0.3% chromium oxide as an indigestible marker.

### Postmortem procedures, sample collection, and analysis

On day 3, collection trays were introduced, and grab samples of fresh excreta were collected for two days and pooled within a cage. Thereafter, 24 hens per group (4 chickens per cage) were randomly selected and euthanized using carbon dioxide asphyxiation for the digesta collection. Ileal digesta was gently flushed with distilled water into labeled plastic containers and stored at −80°C until further analysis for nutrient digestibility.

Representative samples of diets, digesta, and excreta were analyzed for dry matter (DM), calcium, and chromium oxide. DM was determined using the standard AOAC procedure (method 930.15; AOAC 2005). The concentration of chromic oxide was determined following the procedure of Fenton and Fenton [14]. Calcium (method 927.02) concentrations in the feed, the ileal digesta, and excreta were analyzed according to the procedures of the AOAC [15].

### Calculations

The endogenous losses of calcium were calculated as milligrams lost per ingestion of kilogram of feed DM, by using the following formula [16].


(1) $$\text{AID }(\%)=100-([{\text{M}}_{\text{diet}}/{\text{M}}_{\text{digesta}}]\times [{\text{Ca}}_{\text{digesta}}/{\text{Ca}}_{\text{diet}}])\times 100$$

(2) $$\text{BEL }(\text{mg}/\text{kg of DM intake})=({\text{Cr}}_{\text{diet}}/{\text{Cr}}_{\text{digesta}})\times {\text{Ca}}_{\text{digesta}}$$

(3) $$\text{SID }(\%)=\text{AID}+(\text{BEL}/{\text{Ca}}_{\text{diet}})\times 100$$

M_diet_ and M_digesta_ denote the indigestible marker concentrations (mg/kg) in the experimental diet and the ileal digesta samples, respectively. Similarly, Ca_diet_ and Ca_digesta_ indicate the calcium concentrations (mg/kg) in the experimental diet and the ileal digesta samples, respectively. BEL denotes the basal endogenous loss.

### Statistical analysis

The data obtained from the experiment were analyzed using the general linear model procedure for two-way ANOVA to evaluate the main effects (type of diet and site of measurement) in SPSS (Version 26; IBM, Armonk, NY, USA). The experimental unit for this trial was defined as individual birds. Statistical significance was determined at a significance level of p<0.05. Whenever treatment effects were found to be significant (p<0.05), the means were further analyzed and compared using Tukey’s multiple-range test procedures implemented in SPSS software.

## RESULTS

Basal endogenous Ca losses were measured at both the ileal and excretory sites in hens fed a Ca-free diet, while total endogenous losses were assessed using six Ca-sourcing diets (Table 2). At the ileal site, results showed that total endogenous Ca losses were conspicuously higher (p<0.05) in hens fed diets containing various Ca sources compared to those on the Ca-free diet. Among the Ca-supplemented diets, MCP resulted in greater endogenous losses compared to limestone, DCP, and wheat bran. Corn-based diets, however, exhibited relatively lower losses than the other Ca-supplemented diets (p<0.05) in this study. At the excretory site, no significant differences were found in endogenous Ca losses across diets, although values were numerically higher than those observed at the ileal site for each corresponding diet. Moreover, we observed a difference between the two measurement sites (p<0.001), with the excretory site showing higher mean values. Furthermore, the present results were not affected by the experimental diets, although the diet-by-site interactions were noted (p<0.05).

The AID and SID of Ca were compared across various experimental diets. Inorganic Ca sources, including MCP, DCP, and limestone, showed significantly higher digestibility percentages (p<0.05) compared to plant seed-based diets (Table 3). Among the inorganic sources, DCP consistently produced higher AID and SID values, although no significant differences were observed among the inorganic sources themselves. In contrast, diets with corn as the primary feed ingredient for hens showed lower AID and SID values compared to wheat bran and soybean meal. Furthermore, experimental diets had no significant effect on the AID of DM content in this study (Table 3).

## DISCUSSION

Dietary Ca sources influenced Hy-Line Brown layer chicken endogenous loss according to the present study. It may vary depending on the methodology employed, as noted by Anwar and Ravindran [17]. In the present study, we employed consolidated methods, such as a Ca-free diet (purified diet) and a direct method to assess endogenous Ca losses in hens, measuring losses in both the ileal digesta and excreta. We assumed that endogenous calcium losses in the digesta arise from several sources, including saliva [18], gastric secretions [19], bile [20], pancreatic secretions [17], and cellular debris [20] up to the ileum. Although there are no publications specifically addressing the composition of pancreatic calcium content in poultry, it is noted that 40% of the duodenal content consists of pancreatic minerals in pigs [21]. In contrast, Ca losses in the excreta reflect contributions from the urinary and reproductive tracts in addition to the aforementioned intestinal sources [9].

Due to the lack of accurate definitions and measuring reliability, the mechanisms behind endogenous losses are considered the least [22]. While some studies have addressed endogenous Ca losses in broilers [17], there has been no research on endogenous Ca losses in layer chickens. In this scarcity, Adeola et al [23] addressed that endogenous amino acid losses positively correlate with protein concentration. By a similar mechanism, we hypothesize that the observed lower values of endogenous Ca losses in Ca-free diets may be associated with the lack of sufficient Ca in provided diets. Moreover, Ca plays a crucial role in various intestinal fluids, such as digestive enzymes and bile salts (0.30±0.008 g/100 g) [24]. While the precise mechanisms underlying the reduction in endogenous losses remain unclear, it is conceivable that nutrient deficiencies elicit compensatory physiological responses, including reductions in Ca concentration and digestive secretion rates.

Nevertheless, the endogenous Ca losses observed in the current study for Ca-free diets (23.11 mg/kg at the ileum) were lower than the values reported by Anwar and Ravindran [17] (46 mg/kg), despite their use of dried albumen-based diets. This discrepancy could be explained by inherent physiological differences between layer hens and broiler chickens, such as variations in Ca metabolism, nutrient requirements, and gut physiology. According to Carrillo et al [11], calcium bioavailability varies depending on the source. Similarly, our findings revealed differences in the SID values among the various calcium sources tested. We propose that factors such as Ca:P balance regulation, excess Ca excretion, and the formation of insoluble Ca complexes with other minerals (P) could be responsible for the higher total endogenous losses observed in diets containing Ca supplements.

To date, approximately 15 papers have been published since 1974 on Ca digestibility in feed ingredients for broiler chickens [1,25,26], with a few other studies solely focusing on layers [27,28]. In the current study, greater digestibility in inorganic Ca sources could be due to the existence of free ionic form, consistent composition, and rapid dissolution of inorganic Ca in the acidic environment of the digestive tract. In addition, inorganic Ca sources do not contain anti-nutritional factors such as phytates or oxalates, which are present in organic sources and tightly bind Ca, thereby reducing its bioavailability. These properties increase the rapid availability of Ca to laying hens [29,30]. According to published data, Walk et al [31] found no significant difference between the AID and the SID values for different feed ingredients, suggesting minimal endogenous Ca losses. Similarly, David et al [9] emphasized that endogenous losses are negligible compared to the undigested Ca remaining in the gut. In line with previous studies, our results also observed a slight increment in SID values compared to AID without a statistical difference. Furthermore, Kluth and Rodehutscord [32] reported that ileal endogenous losses are associated with the DM intake of the poultry; however, no differences were observed related to the current study.

## CONCLUSION

Diets directly influence the endogenous Ca loss in Hy-Line Brown layer chickens, with inorganic Ca sources leading to greater endogenous losses compared to plant seed-based sources. Additionally, inorganic calcium sources exhibited higher digestibility than plant-based diets in laying hens in the current study. Further investigations into the endogenous loss of laying hens are warranted to validate the findings of the current study, which serves as a pioneering effort in this area of research.

## Figures and Tables

**Table 1:** Ingredients and chemical compositions of the experimental diets (as-fed basis)

Item	Calcium free	Monocalcium phosphate	Dicalcium phosphate	Limestone	Corn	Soybean meal	Wheat bran
Ingredient (%)							
Monocalcium phosphate	-	23.50	-	-	-	-	-
Dicalcium phosphate	-	-	14.29	-	-	-	-
Limestone	-	-	-	10.40	-	-	-
Monosodium phosphate	5.00	-	-	1.29	0.60	1.00	0.07
Corn	-	-	-	-	77.64	-	-
Soybean meal	-	-	-	-	-	38.86	-
Wheat bran	-	-	-	-	-	-	36.55
Soybean oil	2.50	2.50	2.50	2.50	2.50	2.50	2.50
Sucrose	33.56	24.31	28.91	30.21	-	-	-
Cornstarch	33.56	24.31	28.91	30.21	4.58	54.33	46.20
Cellulose	5.00	5.00	5.00	5.00	-	-	-
Dried egg albumen	17.08	17.08	17.08	17.08	11.37	-	11.37
Magnesium oxide	0.20	0.20	0.20	0.20	0.20	0.20	0.20
Sodium bicarbonate	0.30	0.30	0.30	0.30	0.30	0.30	0.30
Sodium chloride	0.40	0.40	0.40	0.40	0.40	0.40	0.40
L-lysine	1.07	1.07	1.07	1.07	1.07	1.07	1.07
DL-methionine	0.74	0.74	0.74	0.74	0.74	0.74	0.74
Vitamin-mineral premix^1)^	0.30	0.30	0.30	0.30	0.30	0.30	0.30
Cr_2_O_3_	0.30	0.30	0.30	0.30	0.30	0.30	0.30
Calculated composition							
Calcium (%)	-	4.00	4.00	4.00	4.70	5.15	4.76
Total phosphorus (%)	0.50	0.50	0.50	0.50	1.01	1.03	0.97
Available phosphorus (%)	0.50	0.50	0.50	0.50	0.58	0.64	0.59
Calcium: available phosphorus	-	8.00:1	8.00:1	8.00:1	8.10:1	8.05:1	8.07:1
Analyzed composition							
Calcium (%)	<0.01	4.08	3.97	4.02	4.81	5.23	4.94
Total phosphorus (%)	0.54	0.49	0.52	0.55	1.22	1.26	1.18

1) Provided per kilogram of diet: vitamin A, 12,000 IU; vitamin D_3_, 3,000 IU; vitamin E, 21 mg; vitamin K_3_, 2,400 mg; vitamin B_1_, 1,200 mg; vitamin B_2_, 4,800 mg; vitamin B_6_, 2,400 mg; vitamin B_9_, 300 mg; D-pantothenic acid, 10,000 mg; nicotinic acid, 15,000 mg; choline, 20 mg; Fe, 24,000 mg from iron sulfate; Cu, 4,500 mg from copper sulfate; Zn, 60,000 mg from zinc oxide; Mn, 72,000 mg from manganese oxide; I, 1,000 mg from potassium iodide; Se, 200 mg from sodium selenite; Co, 150 mg.

**Table 2:** Comparison of the ileal and excreta endogenous Ca losses (mg/kg) in Hy-Line Brown layer chickens estimated using different assay diets

Type of the diet	Site of measurement	Endogenous Ca loss (mg/kg)
Calcium-free (Basal)		23.11^a^
MCP		196.26^b^
DCP		167.04^b^
Limestone	Ileal	195.20^b^
Corn		62.04^a^
Soybean		101.36^ab^
Wheat bran		160.06^b^
p-value		0.008
SEM^1)^		11.311
Calcium-free (Basal)		383.00
MCP		228.98
DCP		208.28
Limestone	Excreta	232.68
Corn		323.97
Soybean		300.12
Wheat bran		412.79
p-value		0.117
SEM^1)^		21.75
The main effect of means		
Type of the diet		
Calcium-free		203.06
MCP		212.62
DCP		187.67
Limestone		213.94
Corn		193.01
Soybean		200.74
Wheat bran		286.43
SEM^1)^		32.44
Site of measurement		
Ileal		129.30
Excreta		298.55
SEM		17.34
p-values		
Type of the diet		0.411
Site of measurement		<0.001
Diet×Site		0.004

Each value represents the mean of six replicates (4 chickens per replicate).

1) Pooled standard error of the mean.

a,b Values with a different superscript within the column differ significantly (p<0.05).

Ca-free, calcium free diet; MCP, mono calcium phosphate-based diet; DCP, dicalcium phosphate-based diet; Limestone, limestone-based diet; Corn, corn based diet; Soybean, soybean meal-based diet; Wheat bran, wheat bran-based diet.

**Table 3:** Comparison of the apparent ileal digestibility (AID) and the standard ileal digestibility (SID) of calcium in different calcium sources in the Hy-Line Brown layer diet

Parameter	Calcium source							SEM^1)^	p-value

	Ca-free	MCP	DCP	Limestone	Corn	SBM	WB		
Calcium AID (%)	-	99.610^c^	99.837^c^	99.557^c^	82.230^a^	92.707^bc^	85.227^ab^	1.834	<0.001
Calcium SID (%)	-	99.630^c^	99.860^c^	99.583^c^	82.253^a^	92.733^bc^	85.247^ab^	1.834	<0.001
Dry matter AID (%)	74.12	74.59	73.43	71.78	72.31	73.03	74.71	1.791	0.324

Each value represents the mean value of six replicates (4 chickens per replicate).

1) Pooled standard error of the mean.

a–c Values with a different superscript within the row differ significantly (p<0.05).

MCP, mono calcium phosphate; DCP, dicalcium phosphate; Corn, corn; SBM, soybean meal; WB, wheat bran.

## References

[1] Lee CW, Kong C (2024). Standardized ileal digestibility of calcium and phosphorus in feed ingredients for 21-day-old broilers. Animals.

[2] Reyer H, Oster M, Ponsuksili S (2021). Transcriptional responses in jejunum of two layer chicken strains following variations in dietary calcium and phosphorus levels. BMC Genomics.

[3] Stein HH, Sève B, Fuller MF, Moughan PJ, de Lange CFM (2007). Invited review: amino acid bioavailability and digestibility in pig feed ingredients: terminology and application. J Anim Sci.

[4] Mutucumarana RK, Ravindran V, Ravindran G, Cowieson AJ (2014). Influence of dietary calcium concentration on the digestion of nutrients along the intestinal tract of broiler chickens. J Poult Sci.

[5] Suttle N (2010). Mineral nutrition of livestock.

[6] Li X, Zhang D, Bryden WL (2017). Calcium and phosphorus metabolism and nutrition of poultry: are current diets formulated in excess?. Anim Prod Sci.

[7] Sinclair-Black M, Garcia RA, Ellestad LE (2023). Physiological regulation of calcium and phosphorus utilization in laying hens. Front Physiol.

[8] Wilkinson SJ, Selle PH, Bedford MR, Cowieson AJ (2011). Exploiting calcium-specific appetite in poultry nutrition. World’s Poult Sci J.

[9] David LS, Anwar MN, Abdollahi MR, Bedford MR, Ravindran V (2023). Calcium nutrition of broilers: current perspectives and challenges. Animals.

[10] Shafey TM (1993). Calcium tolerance of growing chickens: effect of ratio of dietary calcium to available phosphorus. Worlds Poult Sci J.

[11] Carrillo L, Bernad MJ, Monroy-Barreto M, Coello CL, Sumano H, Gutiérrez L (2020). Higher bioavailability of calcium in chickens with a novel in-feed pharmaceutical formulation. Front Vet Sci.

[12] Zhao SC, Teng XQ, Xu DL, Chi X, Ge M, Xu SW (2020). Influences of low level of dietary calcium on bone characters in laying hens. Poult Sci.

[13] Brown HL (c2020). Management guide [Internet].

[14] Fenton TW, Fenton M (1979). An improved procedure for the determination of chromic oxide in feed and feces. Can J Anim Sci.

[15] Association of Official Analytical Chemists (AOAC) (2005). International Official methods of analysis.

[16] Oketch EO, Kim YB, Yu M (2023). Research note: evaluation of standardized ileal amino acid digestibility in feed ingredients for Pekin ducks. Poult Sci.

[17] Anwar MN, Ravindran V (2020). Influence of methodology on the measurement of ileal endogenous calcium losses in broiler chickens. J Appl Anim Res.

[18] Tryon AF, Bibby BG (1966). Preliminary studies on pig saliva. Arch Oral Biol.

[19] Moore JH, Tyler C (1955). Studies on the intestinal absorption and excretion of calcium and phosphorus in the pig: 2. the intestinal absorption and excretion of radioactive calcium and phosphorus. Br J Nutr.

[20] O’Dell BL, Sunde RA (1997). Handbook of nutritionally essential mineral elements.

[21] Zebrowska T, Low AG, Zebrowska H (1983). Studies on gastric digestion of protein and carbohydrate, gastric secretion and exocrine pancreatic secretion in the growing pig. Br J Nutr.

[22] Ravindran V (2021). Progress in ileal endogenous amino acid flow research in poultry. J Anim Sci Biotechnol.

[23] Adeola O, Xue PC, Cowieson AJ, Ajuwon KM (2016). Basal endogenous losses of amino acids in protein nutrition research for swine and poultry. Anim Feed Sci Technol.

[24] Tancharoenrat P, Zaefarian F, Ravindran V (2022). Composition of chicken gallbladder bile. Br Poult Sci.

[25] Anwar MN, Ravindran V, Morel PCH, Ravindran G, Cowieson AJ (2018). Measurement of the true ileal calcium digestibility of some feed ingredients for broiler chickens. Anim Feed Sci Technol.

[26] David LS, Abdollahi MR, Ravindran G, Walk CL, Ravindran V (2019). Studies on the measurement of ileal calcium digestibility of calcium sources in broiler chickens. Poult Sci.

[27] Lichovnikova M (2007). The effect of dietary calcium source, concentration and particle size on calcium retention, eggshell quality and overall calcium requirement in laying hens. Br Poult Sci.

[28] Diana TF, Calderano AA, de Castro Tavernari F, Rostagno HS, de Oliveira Teixeira A, Teixeira Albino LF (2021). Age and calcium sources in laying hen feed affect calcium digestibility. Open J Anim Sci.

[29] Anwar MN, Ravindran V, Walk CL, Kühn I, Stein HH, Kidd MT, Rodehutscord M (2016). Measurement of calcium digestibility in feed ingredients for poultry: methodology and challenges. Phytate destruction: consequences for precision animal nutrition.

[30] Venter KM, Angel R, Fourie J (2024). Determination of calcium and phosphorus digestibility of individual feed ingredients as affected by limestone, in the presence and absence of phytase in broilers. Animals.

[31] Walk CL, Romero LF, Cowieson AJ (2021). Towards a digestible calcium system for broiler chicken nutrition: a review and recommendations for the future. Anim Feed Sci Technol.

[32] Kluth H, Rodehutscord M (2009). Effect of inclusion of cellulose in the diet on the inevitable endogenous amino acid losses in the ileum of broiler chicken. Poult Sci.

